# Pathways to Injury in Chronic Pancreatitis: Decoding the Role of the High-Risk SPINK1 N34S Haplotype Using Meta-Analysis

**DOI:** 10.1371/journal.pone.0002003

**Published:** 2008-04-16

**Authors:** Elie Aoun, Chung-Chou H. Chang, Julia B. Greer, Georgios I. Papachristou, M. Michael Barmada, David C. Whitcomb

**Affiliations:** 1 Division of Gastroenterology, Hepatology and Nutrition, Department of Medicine, University of Pittsburgh, Pittsburgh, Pennsylvania, United States of America; 2 Department of General Internal Medicine, University of Pittsburgh, Pittsburgh, Pennsylvania, United States of America; 3 Department of Biostatistics, University of Pittsburgh, Pittsburgh, Pennsylvania, United States of America; 4 Department of Human Genetics, University of Pittsburgh, Pittsburgh, Pennsylvania, United States of America; Kings College London, United Kingdom

## Abstract

**Background:**

The complex interactions between recurrent trypsin-mediated pancreatic injury, alcohol-associated pancreatic injury and *SPINK1* polymorphisms in chronic pancreatitis (CP) are undefined. We hypothesize that CP occurs as a result of multiple pathological mechanisms (pathways) that are initiated by different metabolic or environmental factors (etiologies) and may be influenced differentially by downstream genetic risk factors. We tested this hypothesis by evaluating the differences in effect size of the high risk *SPINK1* N34S haplotype on CP from multiple etiologies after combining clinical reports of *SPINK1* N34S frequency using meta-analysis.

**Methods and Findings:**

The Pubmed and the Embase databases were reviewed. We studied 24 reports of *SPINK1* N34S in CP (2,421 cases, 4,857 controls) using reported etiological factors as surrogates for pathways and multiple meta-analyses to determine the differential effects of *SPINK1* N34S between alcoholic and non-alcoholic etiologies. Using estimates of between-study heterogeneity, we sub-classified our 24 studies into four specific clusters. We found that *SPINK1* N34S is strongly associated with CP overall (OR 11.00; 95% CI: 7.59–15.93), but the effect of *SPINK1* N34S in alcoholic CP (OR 4.98, 95% CI: 3.16–7.85) was significantly smaller than in idiopathic CP (OR 14.97, 95% C.I. = 9.09–24.67) or tropical CP (OR 19.15, 95% C.I. = 8.83–41.56). Studies analyzing familial CP showed very high heterogeneity suggestive of a complex etiology with an I^2^ = 80.95%.

**Conclusion:**

The small effect of SPINK1 N34S in alcoholic subjects suggests that CP is driven through a different pathway that is largely trypsin-independent. The results also suggest that large effect sizes of SPINK1 N34S in small candidate gene studies in CP may be related to a mixture of multiple etiologic pathways leading to the same clinical endpoint.

## Introduction

Chronic pancreatitis (CP) is a common chronic inflammatory syndrome of the pancreas that has been defined by clinical signs and symptoms linked to end-stage pathological criteria[1]. While acute pancreatitis is recognized as an acute inflammatory response to pancreatic injury [2], the pathophysiological mechanisms underlying the development and progression of CP in humans have yet to be discerned.

CP is defined by the presence of chronic inflammatory cells within the pancreas, progressive fibrosis, sclerosis and parenchymal atrophy [1], [3], [4], [5]. The pancreatic stellate cell (PSC) is the mediator of fibrosis, and is ubiquitous in chronic pancreatitis [6], [7]. The diagnosis of CP requires greater than six month duration of inflammation, permanent loss of exocrine function, and evidence on abdominal imaging studies of duct distortion, fibrosis or calcification. Abdominal pain and diabetes mellitus are clinical features that are also common to CP [1], [3], [4], [5].

CP is sub-classified according to epidemiologic risk factors (e.g. alcoholism, family history, living in certain tropical regions, autoimmune disorders), although the relative risk of recognized environmental factors may be diminutive [3], [8]. If no inciting factor can be identified, CP is termed ‘idiopathic’ and sub-classified as early versus late-onset idiopathic CP by age of diagnosis. Although excessive alcohol consumption is a risk factor for CP, fewer than 5% of alcoholics develop pancreatitis, and many patients with CP do not drink alcohol [8], [9]. Furthermore, the pathophysiological pathways that link the normal pancreas to the end-stage pathology of CP have not been clearly defined.

Several recent human genetic studies have provided insight into components of the pathogenic mechanisms leading to human CP, all of which are related to failure to regulate intrapancreatic trypsin activity and the associated pancreatic injury. The three primary susceptibility genes for CP include the cationic trypsinogen gene (*PRSS1*) [10], the cystic fibrosis transmembrane conductance regulator gene (*CFTR*) [11], [12], and the pancreatic secretory trypsin inhibitor gene, also known as serine protease inhibitor Kazal type 1 (*SPINK1*) [13], [14], [15]. SPINK1 is an acute phase protein whose gene expression and protein concentrations are markedly upregulated by inflammation [16], [17], [18]. *SPINK1* gene mutations are thought to diminish protection against prematurely activated trypsin, and are thereby linked to trypsin-related pancreatic injury [10], [15]. PRSS1, CFTR and SPINK1 seem to play complementary roles in protecting the pancreas from the damage incurred by prematurely activated trypsin.

**Figure 1:**
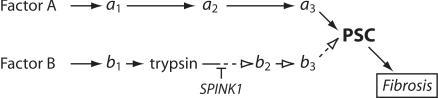
Hypothesis of etiology-defined pathways to pancreatic fibrosis. Hypothetical influence diagram illustrating pathologic pathways linking proximal factor (Factor A and B) to PSC (pancreatic stellate cell) and fibrosis through multiple steps (e.g. a_1_, a_2_, a_3_). Etiological factors of type B activate trypsinogen to trypsin, and therefore their pathologic pathway to the PSC can be interrupted by SPINK1. Etiological factors of type A are independent of trypsin, and therefore will not be influenced by variations in SPINK1 expression or function.

The high-risk *SPINK1* N34S haplotype has been observed in one to three percent of most populations, while the incidence of CP is less than one in ten thousand [14], [19]. Furthermore, the reported range of odds ratios (ORs) describing the risk of *SPINK1* N34S carriers of developing pancreatitis has varied from non-significance [13] to nearly 80 [15]. Wide variations in reported effects of small genetic association studies have been attributed to statistical variation in underpowered studies, poor methodology, publication bias toward studies with large ORs as well as a myriad of other non-biological factors [20], [21], [22]. However, we hypothesize that in the case of the *SPINK1* N34S high-risk haplotype, the variation in reported effect sizes may be a result of differences in the proportion of subjects with pathogenic pathways linking environmental stressors to pancreatic stellate cell (PSC) activation through recurrent trypsinogen activation and inadequate trypsin inhibition by SPINK1. The basic model is illustrated in Figure 1. In this model, SPINK1-regulated pathways would include all upstream etiologic factors associated with recurrent trypsinogen activation (e.g. *PRSS1* and *CFTR* mutations) while SPINK1-independent pathways would include factors that drive PSC to produce fibrosis through mechanisms that are generally independent of recurrent trypsinogen activation (e.g. autoimmune pancreatitis, toxins, pancreatic cancer). Although a number of functionally different risk factors have been statistically associated with alcoholic CP [9], it is not clear if the PSC and fibrosis in alcoholic CP is driven by trypsin-dependent, or independent pathways.

In order to address questions of heterogeneity, and specifically the role of alcohol and *SPINK1* N34S in human CP, we employed meta-analysis in a manner illustrative of the growing realization that meta-analysis is most effective in examining relationships in complex diseases by evaluating between-study heterogeneity [23], [24], [25]. We tested the hypothesis that the true effect size of the functional *SPINK1* N34S haplotype is *pathway-dependent* by reviewing the world literature and gathering data from case-control studies that evaluated the association of *SPINK1* N34S with CP. When possible, we reclassified patients by reported etiology and performed a series of meta-analyses on each category to determine the effect size of the high-risk *SPINK1* N34S haplotype within each etiologic sub-classification. We hypothesized that etiology-based categories defining a trypsin-dependent pathway would be associated with a larger *SPINK1* N34S effect than those that were trypsin-independent.

## Methods

We systematically reviewed the world's literature on *SPINK1* polymorphisms. Only case-control studies were considered. When possible, subjects were classified according to reported etiology, with the following categories as presented in Table 1: Alcoholic chronic pancreatitis (ACP), idiopathic chronic pancreatitis (ICP), familial/hereditary chronic pancreatitis (FCP) and tropical pancreatitis (TP). We then conducted a series of meta-analyses combining subjects within etiology-based sub-classifications as described below.

**Table 1:** Case control studies considered for inclusion in the meta-analyses and patient subclassification within each study.

Author	Year	Journal	Genotyping Method	Population	ACP	ICP	FCP	TCP
Chen et al[13]	2000	J Med Genet	PCR-DGGE	France	Excluded*			
Witt et al[15]	2000	Nat Gen	Direct sequencing	Germany - Austria		+		
Pfutzer et al[14]	2000	Gastro	Direct sequencing	USA - Europac		+	+	
Plendl et al[46]	2001	Am J Med Gen	PCR-RFLP	Germany			+	
Witt et al[47]	2001	JAMA	Direct sequencing	UK –Germany -Switzerland	+			
Rossi et al[30]	2001	Pancreatology	Direct sequencing	Bangladesh	Excluded**			
Chen et al[48]	2001	Gastro	PCR-DGGE	France		+		
Kaneko et al[49]	2001	J Hum Genet	Direct sequencing+RFLP	Japan		+		
Threadgold et al[50]	2002	Gut	Direct sequencing+RFLP	Europac	+	+	+	
Chandak et al[51]	2002	J Med Genet	Direct sequencing	India				+
Drenth et al[52]	2002	Gut	Direct sequencing	Netherlands	+	+	+	
Bhatia et al[53]	2002	Gastro	Direct sequencing	India				+
Hassan et al[54]	2002	Am J Hum Gen	PCR-RFLP	Bangladesh - India				+
Schneider et al[55]	2002	Gastro	Direct sequencing	Bangladesh				+
Audrezet et al[56]	2002	Eur J Hum Gen	PCR-DGGE	France		+		
Truninger et al[57]	2002	Am J Gastro	Direct sequencing	Europe		+		
Schneider et al[58]	2003	Dig Dis Sci	Direct sequencing	USA	+			
Gomez-Lira et al[59]	2003	Eur J Hum Gen	Direct sequencing	Italy		+		
Perri et al[60]	2003	Eur J Hum Gen	Direct sequencing	Italy	+			
Bernardino et al[32] •	2003	JOP	PCR-RFLP	Brazil	+	+	+	
Matsubayashi et al[61]	2003	Cancer Biol Ther	PCR-RFLP	USA		+		
Chandak et al[62]	2004	Gut	Direct sequencing	India	+	+	+	
Lempinen et al[63]	2005	Scand J Gastro	Direct sequencing	Finland	+	+		
Kume et al[29]	2005	Pancreatology	Direct sequencing	Japan	Excluded***			
Lee et al[64]	2005	Dig Dis Sci	PCR-RFLP	Korea	+			
Keiles et al[28]	2006	Pancreas	Direct sequencing	USA	Excluded*			
Shimosegawa et al[65]	2006	J Gastro Hepatol	PCR-RFLP	Japan	+	+	+	
S-Tomaszewska[66] ••	2006	J Pediatr Gastroenterol Nutr	PCR-RFLP	Poland				
Tzetis et al[67]	2007	Clin Genet	PCR-DGGE	Greece		+		
Masamune et al [31]	2007	J Gastroenterol	Direct sequencing	Japan	Excluded****			

* Prevalence of the N34S polymorphism was not reported in the control population.

** Data duplicated in Schneider et al [55].

*** Data duplicated in Shimosegawa et al [65].

**** Data duplicated in Kume et al[29].

• Dropped from the analysis because it did not identify the N34S polymorphism in either the cases or the controls therefore resulting in a null relative weight.

•• Unable to subclassify patients into the mentioned categories due to missing data.

PCR: Polymerase Chain Reaction, RFLP: Restricted Fragment Length Polymorphism, DGGE: Denaturing Gradient Gel Electrophoresis.

### Study selection criteria

The literature search and study review was performed by two separate authors (EA, DCW). Genetic association studies of the *SPINK1* N34S high-risk haplotype in pancreatitis that were published prior to May 2007 were identified by searching the PubMed and the EMBASE databases. Search terms included *polymorphism(s), SPINK1, Serine Protease Inhibitor Kazal type 1, PSTI, N34S* and *pancreatitis*. The reference list of citations in the identified publications was reviewed to identify additional published articles not indexed by the major databases. Genetic association studies that reported the frequency of the *SPINK1* N34S high-risk haplotype in patients with acute and/or chronic pancreatitis and in a control population were selected. When more than one published report used data from the same case and control population, we included only the largest study with extractable data in the meta-analysis. Corresponding authors were contacted in some cases to clarify issues of possible data duplication. Only studies that used validated genotyping methods, such as direct gene sequencing, polymerase chain reaction paired with restricted fragment length polymorphism and denaturing gradient gel electrophoresis, were included. Studies based on linkage results (pedigree studies), case reports, editorials, review articles and studies published in a language other than English were excluded.

### Data Abstraction

Data abstraction was performed by two separate authors (EA, CC) and differences were resolved by discussion. The data elements included the first author, journal, year of publication, country of origin, racial background of the study population (when mentioned), demographics, reported etiology of CP (alcoholic, idiopathic, familial, tropical, etc) and the number of cases and controls. Allelic frequency, genotypic distribution and genotyping methods were recorded. All statistical analysis was based on the number of alleles–as opposed to number of patients, in order to better quantify homozygous cases.

### Statistical Methods

For each study, we initially evaluated the association of *SPINK1* N34S high-risk haplotype with CP separately. A preliminary meta-analysis combining all studies regardless of etiology was conducted. We then conducted a series of meta-analyses assessing the degree of risk of various categories of CP with *SPINK1* N34S. Effect size was expressed as an OR with the corresponding 95% confidence interval (CI). Heterogeneity between studies was tested using the Cochran Q statistic and the I^2^ value. Because all statistical tests for heterogeneity are weak, we also included the 95% confidence interval for I^2^ [26], which was calculated based on previously described methods[27]. When heterogeneity was not overtly evident, we performed the meta-analysis using both the fixed-effect (Mantel-Hanszel method) and the random-effect models (DerSimonian and Laird method). When heterogeneity was present, we only reported the results using the random-effect model. The Mantel-Hanszel (MH) method was selected over other fixed-effect methods because of potential small sample sizes of mutation carriers. An I^2^ value of 50 was considered the threshold, above which studies were considered too heterogeneous to be combined. The Haldane continuity correction (adding 0.5 to each cell) was used if the quantity of N34S-containing genotypes was equal to zero in either the cases or the controls. The presence of publication bias and small study effects were assessed using the Egger regression asymmetry test for funnel plot and the Begg-Mazumdar adjusted rank correlation test. Statistical significance was considered at a p-value of ≤0.05. In cases where the polymorphism was not detected in either cases or controls, the study was omitted from the meta-analyses as it carried a relative weight of zero. All meta-analyses were conducted using Stata version 8.2 (Stata, Inc., College Station, Texas) and Comprehensive Meta-Analysis version 2.0 (Biostat Inc. Englewood, New Jersey).

## Results

**Figure 2:**
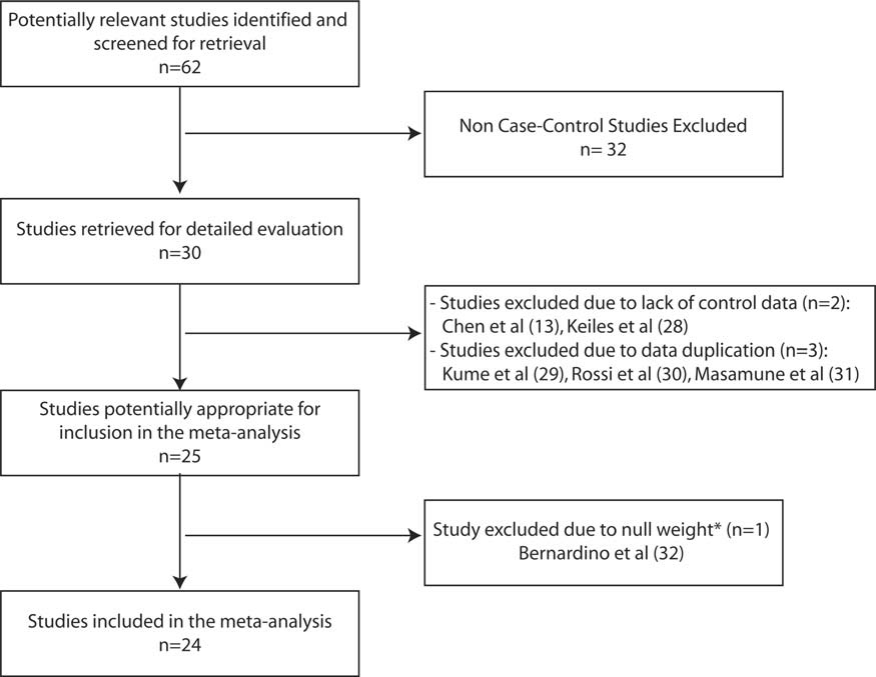
Flow diagram of the studies included in the meta-analysis. * The report by Bernardino et al was excluded from the meta-analysis because it did not detect the N34S haplotype in neither the cases nor the controls and was therefore assigned a weight of zero.

Figure 2 is a flow diagram illustrating the studies included. Thirty case-control studies evaluating the association of the *SPINK1* N34S polymorphism with CP were identified (Table 1). Two studies were excluded because the prevalence of the polymorphism was not reported in the control population [13], [28]. An additional three studies were excluded due to data duplication in other publications [29], [30], [31]. Additionally, The report by Bernardino et al [32] was excluded from the analysis because it did not identify the N34S polymorphism in either cases or controls. A total of 24 studies were therefore evaluated.

### Chronic Pancreatitis–All etiologies combined

**Figure 3:**
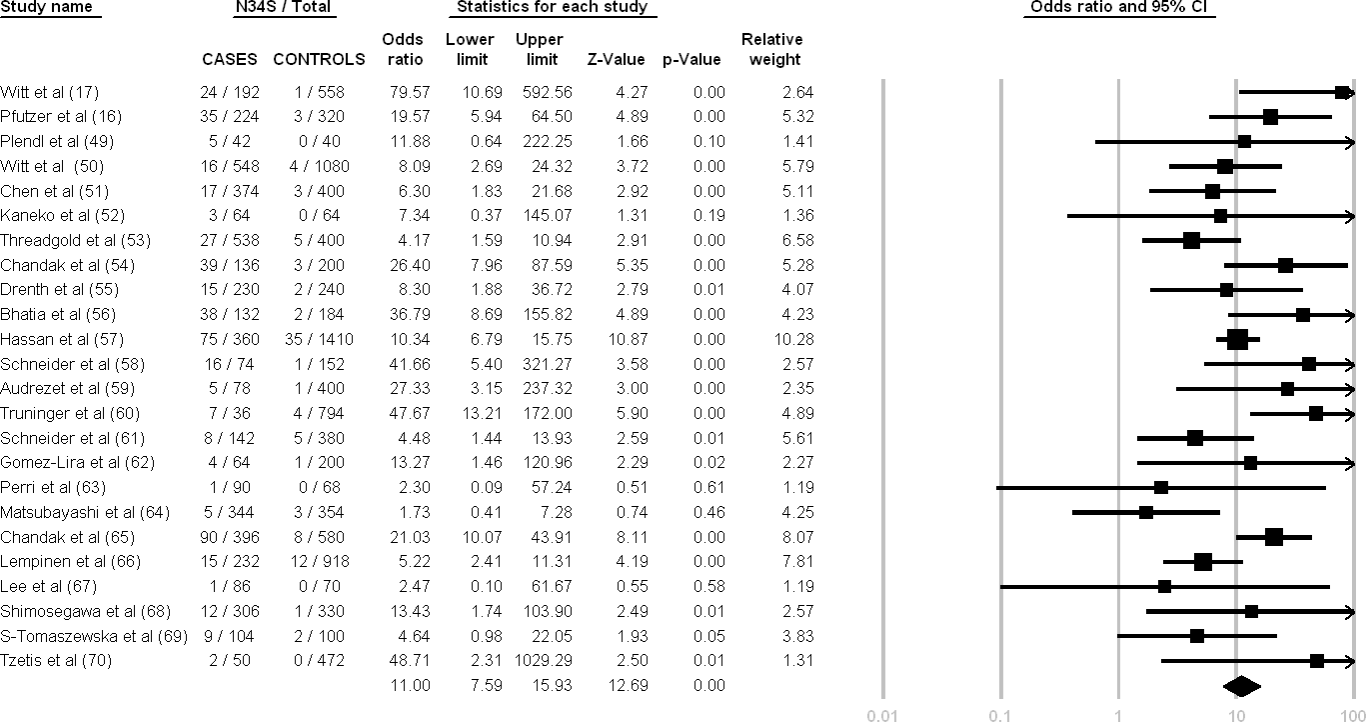
Meta-analysis results for chronic pancreatitis all etiologies combined based on allelic frequency. Heterogeneity testing: Q-value = 41.05, df = 23, p = 0.01, I^2^ = 43.97 (95% CI: 10.56–64.90).

Figure 3 summarizes the results of the initial meta-analysis with all etiologies of CP combined. The total number of patients from these studies was 2,421 with 4,857 controls. The mutation was detected in 469 of 4,842 patient alleles and in 96 of 9,714 control alleles. Significant heterogeneity was detected (Q = 41.05, df = 23, p = 0.01, I^2^ = 43.97%). The random effect model showed a combined OR of 11.00 (95% C.I. = 7.59–15.93). Both the Egger and the Begg-Mazumdar tests were not statistically significant with p = 0.65 and p = 0.94 respectively.

### Alcoholic Chronic Pancreatitis

**Figure 4:**
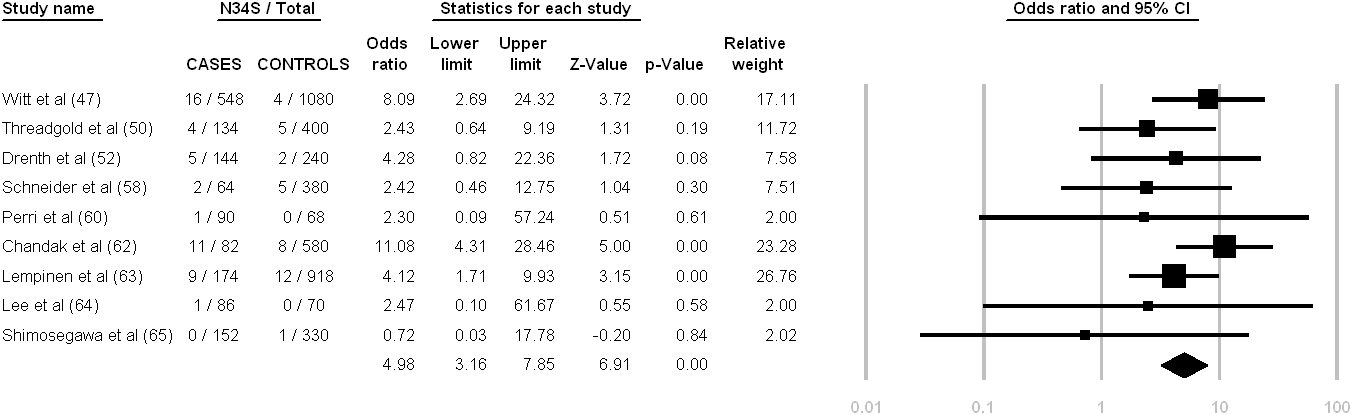
Meta-analysis results for alcoholic chronic pancreatitis based on allelic frequency. Heterogeneity testing: Q-value = 7.36, df = 8, p = 0.5, I^2^ = 0 (95% CI: 0.00–62.01).

We identified patients with alcoholic CP from 9 studies (737 patients, 2,033 controls). Overall, alcohol was the etiologic factor leading to chronic pancreatitis in 31% of the patients that we were able to classify. Six of nine studies failed to identify a statistically significant association between the *SPINK1* N34S polymorphism and alcoholic CP. The *SPINK1* N34S high-risk haplotype was reported in 49 of 1,474 patient alleles and in 37 of 4,066 control alleles. There was no heterogeneity detected (Q = 7.36, df = 8, p = 0.50, I^2^ = 0%). Both the fixed and the random-effect model showed a pooled OR of 4.98 (95% C.I. = 3.16–7.85)–the lowest among the different categories that we analyzed. Figure 4 summarizes the meta-analysis results pertaining to patients with alcoholic CP.

### Tropical Chronic Pancreatitis

**Figure 5:**
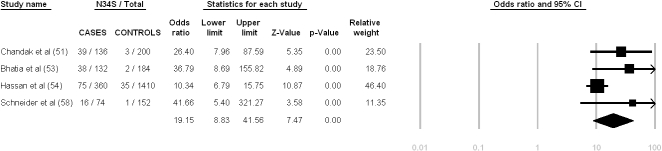
Meta-analysis results for tropical pancreatitis based on allelic frequency. Heterogeneity testing: Q-value = 5.72, df = 3, p = 0.13, I^2^ = 47.56 (95% CI: 20.96–78.99).

Four studies assessed patients with tropical pancreatitis (351 patients, 973 controls). The high-risk haplotype was detected in 168 of 702 patient alleles and in 44 of 1,946 control alleles. The heterogeneity testing showed Q = 5.72, df = 3, p = 0.13, I^2^ = 47.56%. The pooled OR calculated using the random-effect model was 19.15 (95% C.I. = 8.83–41.56). Figure 5 summarizes the meta-analysis results pertaining to patients with tropical pancreatitis.

### Idiopathic Chronic Pancreatitis

**Figure 6:**
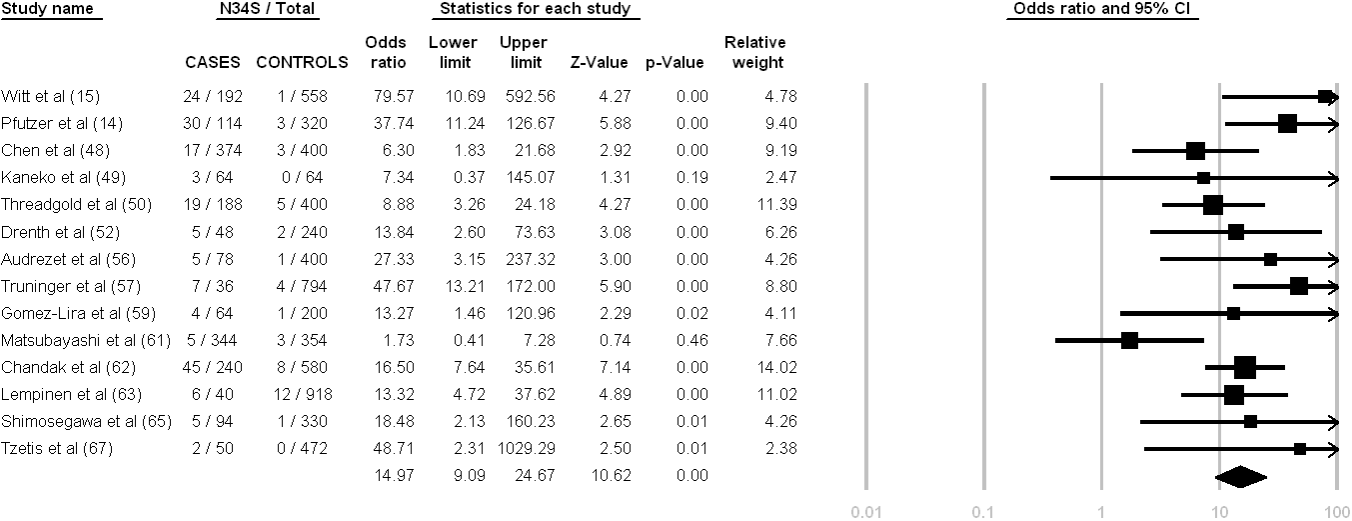
Meta-analysis results for idiopathic chronic pancreatitis based on allelic frequency. Heterogeneity testing: Q-value = 20.86, df = 13, p = 0.08, I^2^ = 37.98 (95% CI: 0.00–65.00).

Fourteen studies were included in this analysis (963 patients, 3,015 controls), only two of which did not detect a statistically significant association with *SPINK1* N34S (figure 6). The Cochran's Q statistics was calculated at 20.86 with a p value of 0.08 and an I^2^ of 37.98%. The pooled OR was 14.97 (95% C.I. = 9.09–24.67).

### Familial and Hereditary Chronic Pancreatitis

Six studies (249 patients, 955 controls) were analyzed. Significant heterogeneity was detected by the Cochran Q statistic (Q = 26, df = 5, p = 0.00). Additionally, the I-squared value was 80.94%. Due to the high degree of heterogeneity, these studies were not combined.

## Discussion

As stated in a recent article on meta-analysis: “It is well appreciated now that besides estimating summary effects, estimation and, if possible, explanation, of the between-study heterogeneity is a very important goal for meta-analysis”[24]. Furthermore, the article goes on to state that “in the presence of between-study heterogeneity in the genetic effects, there may be important implications for the interpretation of the results”. The aim of the current study was to understand the associations between alcohol, the high-risk *SPINK1* N34S haplotype, and CP, and to assess the strength of any association using etiology-based classifications from previously reported studies using meta-analyses. As expected [20], [21], we observed wide variation in ORs among small studies which was reflected in the global meta-analyses results (Figure 3). Stratifying subjects by commonly reported etiologies and performing subset meta-analysis to determine the specific effects of *SPINK1* N34S in subpopulations, in some cases, revealed low variance. This finding suggests that these subpopulations are associated with more homogeneous pathological mechanisms. The different ORs and, occasionally, non-overlapping CIs (e.g. alcohol versus idiopathic, tropical etiologies) suggest that the CP syndrome encompasses several patient subpopulations, and may indicate that the pathologic pathway linking the proximal etiology to the PSCs in fibrosing CP are regulated to different degrees by the activity of SPINK1, as envisioned in Figure 1.

When meta-analysis is applied to complex genetic syndromes, the measured effect size of a genetic variable between studies is often widely discrepant [20], [21]. A way to interpret the observation that very large effect sizes in small genetic studies are often not “reproducible” in subsequent studies has been attributed to a combination of false-positive findings (e.g. related to multiple testing without correction) and publication bias toward reports with large effect sizes.

Another potential explanation is that there are multiple sub-populations with different complex, multi-step etiologic pathways that all lead to the same phenotype (e.g. end-stage organ fibrosis). Thus, a genetic factor that is critical in one etiologic pathway will only be shown to have a large effect size in populations in which that pathways dominates [25], [33]. In other populations, in which the dominant etiological pathway is independent of the gene variant in question, the measured effect size of the genetic variant may be small.

Alcohol is a toxin that damages the pancreas by altering key regulatory processes, causing direct injury to acinar cells and driving stellate cells to produce fibrosis. Specifically, studies have shown that alcohol can act directly on the brainstem [34], acinar cells [35], immune system [36], and PSCs [37]. The association of *SPINK1* N34S with the risk of alcoholic CP was significant, but the lowest of the subgroups. This observation suggests that while a portion of the insult may be associated with premature trypsinogen activation, alcohol's primary effects seem to be via *SPINK1-* independent pathways (similar to Factor A in Figure 1), and largely through the direct effects on the immune system and stellate cells, as has been demonstrated in animal experiments [37], [38], [39].

The pathway leading from an unknown factor in tropical pancreatitis to PSC activation and fibrosis is more strongly affected by *SPINK1* N34S then was seen in alcoholic CP. Although the proximal, presumably trypsin-associated factors are unknown, Mahurkar and colleagues have recently reported a higher incidence of mutations in the cathepsin B gene (*CTSB*) in patients with TP than controls [40]. Cathepsin B is a lysosomal enzyme present in pancreatic acinar cells that appears to be important in protecting the pancreas from premature trypsinogen activation [41], [42], suggesting that *CTSB* mutations may increase the risk of inappropriate trypsin activity within the acinar cells. This may be one of several critical factors contributing to the etiology of TP.

Patients with idiopathic CP had a significant association with the *SPINK1* N34S polymorphism as evidenced by a pooled OR of 14.66. Noone et al. reported a high incidence of *CFTR* mutations in patients with ICP and further demonstrated that the combined risk of *CFTR* mutations and *SPINK1* polymorphisms was multiplicative rather than additive [43]. Although the various etiologies of idiopathic CP are largely unknown, the significant enrichment of this group with subjects carrying *PRSS1* and *CFTR* mutations and the large effect of *SPINK1* polymorphisms with progression to CP is consistent with our proposed model. Of note, the highest reported OR of any study (OR = ∼80) was that of Witt et al that was conducted in a relatively homogenous group of children with early onset idiopathic CP where few, if any, environmental exposures played a role, especially tobacco smoking and alcohol [15]. Thus, the high OR reflected an enrichment of the pathway that is most strongly regulated by SPINK1 rather than the random effects of chance.

The risk of familial and hereditary CP is strongly associated with *SPINK1* N34S, but these data should be interpreted with caution. Hereditary pancreatitis is an autosomal dominant disorder with very high penetrance that is caused by gain-of-function mutation in the cationic trypsinogen gene [10]. The etiology of hereditary pancreatitis is unequivocally linked to trypsin-associated pathway of recurrent acute pancreatitis leading to CP, but the effect is to such a degree that normal expression of SPINK is not sufficient to prevent it. On the other hand, a major proportion of familial chronic pancreatitis that is not autosomal dominant is associated with multiple family members with homozygous *SPINK1* mutations. In this case, the etiology-based classification system is biased toward an association with *SPINK1*. These factors likely contribute to the high heterogeneity of effects in this classification.

The current study has several limitations. One of the challenges in understanding CP in human subjects is distinguishing trypsin-dependent and trypsin-independent pathways in CP from the central role of trypsin in acute pancreatitis. Indeed, AP may be necessary to initiate CP by activating the immune system within the pancreas (including PSCs), thereby initiating the fibrosing process [44], [45]. Thus, although CP is driven by trypsin-depended and trypsin- independent factors, the fact that an individual is initially susceptible to the first episode of AP may blur the distinction between trypsin-dependent and trypsin-independent pathways to CP. Another limitation is that the published reports did not include or classify pancreatic fibrosis caused by autoimmune pancreatitis or pancreatic cancer, which, because they are thought to be trypsin-independent, would have been an important comparison group for *SPINK1* N34S effects. Furthermore, the effect of ethnicity and race was usually not reported, although the country of origin was available (Table 1) and the etiologies of interest were clearly reported. Genetic polymorphisms often vary by race or ethnicity. Further studies are warranted to more thoroughly evaluate racial and/or ethnic variation in SPINK polymorphisms. Another limitation relies in the fact that classification of the patients into etiology-based subgroups was carried based on the etiologies listed in the manuscripts included in the analysis. It is unlikely that all twenty-four centers used the same criteria to diagnose and categorize these patients and therefore variations in the diagnostic criteria may explain some of the heterogeneity observed. Furthermore, a certain degree of misclassification may have occurred as a result and there is considerable residual heterogeneity beyond what the etiologic grouping can explain. Additionally, the presence of potential confounding variables or modifying factors could not be completely ruled out due to the limited amount of information and data assessing such factors in each study. Despite these challenges, clear differences in the effect of SPINK1 in different etiologies were observed.

In conclusion, meta-analysis of association studies examining the *SPINK1* N34S polymorphism in CP confirms a significant overall pathologic association, although the reported effect size varies significantly depending on the etiology of CP. Modeling CP as a complex syndrome resulting from various pathogenic pathways with or without recurrent trypsinogen activation allows for the direct assessment of the effect of *SPINK1* polymorphisms in the development of CP. While the subgroup analysis needs to be interpreted with caution, our results suggest that much of the variance in reported ORs between small studies of candidate genes in complex disorders may be attributed to a mixture of multiple etiologic pathways leading to a single clinical endpoint (e.g. organ fibrosis in CP). Alcohol appears to drive fibrosis in alcoholic CP primarily through a trypsin-independent pathway as reflected by the significantly lower association of *SPINK1* N34S with this etiology. Additional studies are warranted to further assess the presence of any confounding or modifying factors and to elucidate the various pathophysiologic mechanisms involved and their implications in the etiology of CP.
